# Overwintering of Rabies Virus in Silver Haired Bats (*Lasionycteris noctivagans*)

**DOI:** 10.1371/journal.pone.0155542

**Published:** 2016-05-19

**Authors:** April D. Davis, Shannon M. D. Morgan, Michelle Dupuis, Craig E. Poulliott, Jodie A. Jarvis, Rhianna Franchini, Anne Clobridge, Robert J. Rudd

**Affiliations:** 1 Rabies Laboratory, Wadsworth Center, New York State Department of Health, Slingerlands, New York, United States of America; 2 Department of Biological Sciences, University of Albany, State University of New York. Albany, New York, United States of America; 3 Academy of Holy Names, 1075 New Scotland Rd, Albany, New York, United States of America; Thomas Jefferson University, UNITED STATES

## Abstract

Silver-haired bats, (*Lasionycteris noctivagans*) are semi-colonial, migratory tree bats that have infrequent contact with humans. Despite the species rarity, the *L*. *noctivagans* rabies variant is the most commonly reported rabies virus variant (RABV) in domestically acquired human rabies cases in the US. Unlike big brown bats (*Eptesicus fuscus*) and little brown bats (*Myotis lucifugus*), *L*. *noctivagans* are not considered true hibernators. It is unknown if RABV can overwinter in hibernating *L*. *noctivagans* or is only maintained in members of this taxa that migrate to warmer climates. To better understand RABV overwintering in this species, *L*. *noctivagans* were inoculated intramuscularly with either a homologous RABV (*L*. *noctivagans* Virus 1) or one of two heterologous RABV (*Eptesicus fuscus* Virus 2 and *Myotis lucifugus* Virus 1). Five days following inoculation, *L*. *noctivagans* were placed in a hibernation chamber for 6 weeks. Our results demonstrate that rabies virus can overwinter in *L*. *noctivagans* yet the incubation period was extended 6 weeks when compared to bats maintained at ambient temperatures. Additionally, we found that the longer the incubation period, the greater the viral dissemination to the salivary glands. Similar to our previous studies, *L*. *noctivagans* were most susceptible to a homologous variant. In summary, we found that RABV incubation is extended following a subcutaneous exposure or maintenance in hibernation and longer incubation times increase dissemination and potential for transmission.

## Introduction

Rabies virus (RABV) can be found in wild animals year round, yet infections increase substantially during certain times of the year. In the Northeastern US, the percentage of rabies positive animals increases from June through October [1]. The increase coincides with warmer weather, bats moving to summer roosts and nursery colonies, and increased activity of wildlife. Warmer weather also results in more human outdoor activity and thus greater likelihood for interaction between the general public and wildlife.

In most of the United States, big and little brown bats, *Eptesicus fuscus* and *Myotis lucifugus*, respectively, enter torpor in late September and arouse early to mid-April [2]. Tree bats, including *L*. *noctivagans*, migrate during late August through early September [3]. Mating swarms are common shortly before hibernation, increasing the interaction and potential rabies exposure among bats. A female may mate with multiple males prior to hibernation and contact with saliva is common as biting often occurs during the mating process [2].

Not all bats hibernate; some, such as Mexican Free-tailed bats (*Tadarida brasiliensis*), migrate south for the winter. Other species, including Silver-haired bats (*L*. *noctivagans)*, Hoary bats (*Lasiurus cinereus*), and Red Bats (*Lasiurus borealis*) are both migratory and quasi-hibernators [2]. Unlike true hibernators such as *E*. *fuscus* and *M*. *lucifugus*, tree bats such as *L*. *noctivagans*, *L*. *cinereus*, and *L*. *borealis* will migrate south and enter torpor for varying length of time, usually in tree cavities, bark crevasses, and leaf litter. Although tree bats hibernate, it may be interrupted with periodic arousals and movement to a new hibernaculum.

Little is known concerning the effects of hibernation on rabies virus infection and transmission in bat populations. Significant physiological changes occur during hibernation including decreased immune function and metabolism [4, 5]. As cellular metabolism slows, a concurrent reduction in viral replication may occur. Rabies virus replicates utilizing cellular machinery and may suffer due to the decreasing host metabolism and lack of cellular material available for the virus. However, down regulation of the immune system may allow the virus to remain undetected by the immune system for a greater length of time.

In this report, we describe rabies virus maintenance and pathogenesis in *L*. *noctivagans* bats following experimental inoculation, hibernation, and subsequent arousal. To evaluate differences among homologous and heterologous RABV during hibernation, *L*. *noctivagans* were inoculated with the homologous RABV or one of the two heterologous RABV. This allowed us to better understand incubation times, dissemination, and transmissibility following hibernation. Our results demonstrate that RABV can overwinter in bats and the increase incubation allows for greater potential for transmission in the spring and summer.

## Materials and Methods

### Ethics Statement

Experimental design and animal care were done in compliance with the USDA Animal Care and Welfare Act (AWA) and the Association for Assessment and Accreditation of Laboratory Animal Care International (AAALAC). The use of bats in this experiment was approved and conducted in accordance with the Wadsworth Center IACUC.

### Animals

Forty-five adult, mixed gender *L*. *noctivagans* bats were quarantined within a Biosafety level 3 facility for at least 3 months. Upon entering the colony, all bats were identified with a uniquely colored wing band, weighed, and an oral swab was collected and tested for the presence of RABV RNA via real-time RT-PCR.

Bats were provided fresh water and ad-lib gut-loaded mealworms daily [6]. The ambient temperature and humidity were maintained at 24-270C and 70–88%, respectively. Twice weekly bats were given a brief physical exam, weighed, and an oral swab was collected. Bats losing more than 0.5 g or more between examinations were reweighed daily. If weight loss continued but behavior was normal, the bat was placed in an isolation cage, closely monitored, fed 0.5–1.0 ml beef baby food (Gerber^®^ Florham Park, NJ) and if necessary, administered 0.5ml lactated ringers saline subcutaneously every 24 hours. If a bat did not improve or demonstrated clinical signs of rabies, it was euthanized and tested for rabies via the direct fluorescent antibody test (DFA); if rabies positive, conventional PCR was performed to determine the infecting RABV.

Two weeks following introduction into the colony, bats were bled to determine the presence or absence of viral neutralizing antibodies inoculated (VNA). All bats were seronegative for RABV VNA (Tables 1 and 2). One week prior to inoculation, bats were placed into mixed gender groups of 5. Bats that were maintained at ambient temperatures for the duration of the experiment were inoculated either subcutaneously (SC) or intramuscularly (IM) with 10μl of 10^4^TCID_50_ of bat-origin rabies virus using one of three variants: *L*. *noctivagans* (LnV1), *Myotis lucifugus* (MlV1), or *Eptesicus fuscus* (EfV2). Bats in the hibernation groups were inoculated IM with one of the three variants as described previously. Bats receiving an intramuscular inoculation were inoculated in the right deltoid muscle. Bats inoculated subcutaneously were inoculated in the subcutaneous tissues superficial to the right deltoid muscle.

**Table 1:** Bats maintained in hibernation for 6 weeks. Serological and survival results of *L*. *noctivagans* inoculated with homologous or heterologous RABV via SC or IM inoculation. Regardless of the virus or route, the titer of inoculum was 10^4^TCID_50_.

Bat group and no.	Virus/ route	Incubation time (dpi)^a^	Incubation time following arousal^b^	qPCR results for oral swabs (dpi)^c^	Ct value of oral swab	Status of bat	Infecting Variant	Rabies virus titer VNA (IU/ml) days post primary inoculation primary inoc			
Group 1	LnV1/IM							Pre inoc^d^	14 days post hibernation	30 days post hibernation	45 days post challenge^e^
1				N		A		≤LOD	≤LOD	2.0	≤LOD
2		50	27(18)	P (45)	29	D	LnV1	≤LOD	0.525	2.0^g^	
3		66	19(42)	N	>40^i^	D	LnV1	≤LOD	4.0^g^		
4		80	33 (42)	P (71)	29	D	LnV1	≤LOD	≤LOD	≤LOD^g^	
5				N		A		≤LOD	≤LOD	≤LOD	≤LOD
Group 2	EfV2/IM										
6				N		A		≤LOD	ND^h^	>9.6 IU	6.2
7				N		A		≤LOD	≤LOD	≤LOD	≤LOD
8		137	90(42)	P (136)	30	D	EfV2	≤LOD	≤LOD	>16 IU^g^	
9				N		A		≤LOD	≤LOD	2.8	8.8
10				N		A		≤LOD	≤LOD	≤LOD	1.7
Group 3	MlV1/IM										
11		70	23 (42)	N	>40	D	MlV1	≤LOD^f^	≤LOD	≤LOD^g^	
12				N		A		≤LOD	≤LOD	≤LOD	3.1
13				N		A		≤LOD	≤LOD	≤LOD	4.6
14				N		A		≤LOD	8.3	4	≤LOD
15				N		A		≤LOD	≤LOD	≤LOD	≤LOD

ND Not done; serum sample was inadequate for testing

^a^ Days post inoculation

^b^ Negative or positive (dpi oral swabs were positive)

^c^ The survival status of the bat is denoted A for alive and D for deceased

^d^ Bats were bled prior to rabies inoculation

^e^ Days post challenge. Bats were challenged 9 mo following the first inoculation

^f^≤LOD = Below the limit of detection

^g^ denotes terminal bleed

^h^ This bat was caught more than 150 days before clinical signs developed. Site of inoculation unknown

^i^. Ct values >40 are considered negative

**Table 2:** Bats maintained at ambient temperature. Serological and survival results of *L*. *noctivagans* inoculated with homologous or heterologous RABV via SC or IM inoculation. Regardless of the virus or route, the titer of inoculum was 10^4^TCID_50_.

Bat group and n	Virus/route	Incubation time (dpi)^a^	qPCR results for oral swabs (dpi)^b^	Ct value of oral swab	Status of bat^c^	Infecting Variant	Rabies virus titer VNA (IU/ml) days post primary inoculation			
Group 1	LnV1/IM						Pre inoc^d^	90dpi	60 dpc^e^	180dpc
16			N		A		≤LOD^f^	≤LOD	ND	≤LOD
17		22	N	>40^i^	D	LnV1	≤LOD	≤LOD ^g^		
18			N		A		≤LOD	≤LOD	ND	≤LOD
19		20	N	>40	D	LnV1	≤LOD	≤LOD ^g^		
20		14	N	>40	D	LnV1	≤LOD	1.0 ^g^		
Group 2	LnV1/SQ									
21			N		A		≤LOD	≤LOD	≤LOD	≤LOD
22		33	P (30)	32	D	LnV1	≤LOD	0.25 ^g^		
23			N		A		≤LOD	≤LOD	≤LOD	≤LOD
24		14	P (14)	31	D	LnV1	≤LOD	2.0 ^g^		
25			N		A		≤LOD	≤LOD	ND	
Group 3	EfV2/IM									
26			N		A		≤LOD	≤LOD	≤LOD	≤LOD
27			N		A		≤LOD	≤LOD	≤LOD	≤LOD
28			N		A		≤LOD	1.3	≤LOD	≤LOD
29			N		A		≤LOD	2.1	0.5	0.5
30			N		A		≤LOD	≤LOD	≤LOD	≤LOD
Group 4	EfV2/SC									
31			N		A		≤LOD	5.0	1.2	0.8
32		33	P (29,32)	28,28	D	EfV2	≤LOD	0.25 ^g^		
33			N		A		≤LOD	1.0	3.4	0.45
34			N		A		≤LOD	≤LOD	ND	ND
35		37	P (37)	31	D	EfV2	≤LOD	≤LOD ^g^		
Group 5	MlV1/IM									
36			N		A		≤LOD	≤LOD	≤LOD	≤LOD
37		≥150^h^	N	>40	D	LnV1	≤LOD	1.25 ^g^		
38			N		A		≤LOD	≤LOD	0.3	≤LOD
39			N		A		≤LOD	≤LOD	ND	≤LOD
40			N		A		≤LOD	2.0	≤LOD	≤LOD
Group 6	MlV1/SC									
41			N		A		≤LOD	≤LOD	ND	0.38
42			N		A		≤LOD	2.6	ND	≤LOD
43			N		A		≤LOD	≤LOD	≤LOD	1.4
44			N		A		≤LOD	≤LOD	2.6	3.15
45			N		A		≤LOD	≤LOD	≤LOD	≤LOD

ND Not done; serum sample was inadequate for testing

^a^ Days post inoculation

^b^ Negative or positive (dpi oral swabs were positive)

^c^ The survival status of the bat is denoted A for alive and D for deceased

^d^ Bats were bled prior to rabies inoculation

^e^ Days post challenge. Bats were challenged 9 mo following the first inoculation

^f^≤LOD = Below the limit of detection

^g^ denotes terminal bleed

^h^ This bat was caught more than 150 days before clinical signs developed. Site of inoculation unknown

^i^. Ct values >40 are considered negative

To simulate exposure during mating swarms and hibernation, bats in the hibernation study were maintained at ambient temperature and humidity for 5 days following inoculation. On the sixth day, bats were placed within a cage inside the hibernation chamber. The cage in which the bats were placed was a 24” L x 13.5’ W x 11” H lucite plexiglass enclosure. Although the exact cage used in this experiment has been discontinued, it is similar to the Macaw Crystal Shuttle Bird Travel Carrier (Crystal Flight, USA). The cage contained multiple air holes approximately 2 cm in diameter. Plastic black pet screen (Phifer, USA) was attached onto the interior walls of the cage to allow bats to utilize all the vertical area of the cage and provide roosting areas. The pet screen also covered the air holes to prevent bat injury or escape. Disposable absorbent bench liner pad was cut to fit the bottom of the cage. Although water was provided, food was not. In a previous pilot hibernation experiment, bats would arouse from torpor every night if food was available in the cage, thus defeating our experiential design of a true hibernation experiment.

An infrared motion detecting video camera (PIR Smart Cam Spyville.com, TN) was placed over the top of the cage to visualize and record any activity that occurred during hibernation. The videos were recorded on a laptop and viewed daily by 9am. The system was rechecked at 5pm to ensure no movement occurred during the day.

The hibernation chamber was maintained at 7.0°C and 85–90% RH. Temperature and humidity data were indicated by the LED screen on the chamber as well as independent data monitors placed within the chamber. Temperature and humidity remained stable throughout the experiment.

Our goal was maintain bats in the hibernation chamber for six weeks. Based on previous research, bats were expected to arouse once every 10–14 days for 30–120 minutes [7]. Any bat that was active more than 4 nights in a row was removed from the colony, weighed, and returned to their home cage at ambient temperature and humidity as described above. Bats were removed after 4 days because frequent arousal and activity at hibernation temperature requires a considerable amount of energy and bats can starve if food is not available. If a bat had lost more than 5 grams, it was hand fed a small amount of beef baby food. Bats removed from the hibernation chamber were weighed daily to ensure weight gain.

After six weeks, the hibernation chamber was slowly brought to ambient temperature and humidity over the course of 8 hours. Bats were removed from the hibernation chamber, weighed, and returned to their home cage. If the weight loss was 5 g or greater, bats were fed as described previously. Ad lib mealworms and water were provided. The bats were rechecked 4 hours after removal from the hibernation chamber to ensure bats were active and appeared healthy. Bats were weighed daily for 7 days then biweekly, at which time an oral swab was also collected.

All surviving bats were inoculated a second time IM with 10^4^ TCID_50_ LnV1 in the right deltoid muscle 9 months after the first inoculation. Animals were monitored at least twice a day and weighed twice a week when orals swabs were collected. Bats were bled via the uropatagial vein at time points as noted in Tables 1 and 2. Any bat that demonstrated clinical signs of rabies was immediately euthanized, bled, and necropsied. Multiple tissues were collected during necropsy and processed for real-time RT-PCR.

None of the bats died of rabies or of any other complication during hibernation. The ability to monitor the activities of the animals was invaluable to the success of this project. To allow bats to remain in the hibernation chamber that did not go into torpor would have resulted in the starvation of three bats. This was considered both inhumane and unacceptable to our laboratory and IACUC. The addition of an infrared motion activated video camera was a cost effective way to improve the husbandry of hibernating bats. It also provided additional insight into the duration of torpor bouts, type of activities that occur during hibernation, and regular arousal periods.

### Viruses

All viruses were isolated from the salivary glands of homologous species, eg the *L*. *noctivagans* RABV (LnV1) was isolated from a *L*. *noctivagans* submitted to the rabies laboratory for routine rabies testing. Both EfV2 and MlV1 were isolated from the salivary glands of *E*. *fuscus* and *M*. *lucifugus*, respectively. All isolates were passaged three times in neuroblastoma cell culture and a TCIT_50_ was performed to determine the titer of each variant.

### Serology

To evaluate the presence of anti-rabies viral neutralizing antibodies (VNA), bats were bled from the uropatagial vein five weeks prior to inoculation to ensure seroconversion had not occurred. The rabies tissue culture serum neutralization test protocol was modified to reduce the amount of sample required to 25ul as previously described [8].

Bats maintained at ambient temperatures were bled 90 days after the first inoculation. Bats maintained in the hibernation chamber were bled two and four weeks after removal from the hibernation chamber. The three bats that were removed prior to the 6-week end date were bled two and four weeks following their return to ambient temperatures.

All surviving bats were challenged 9 months following the first inoculation of RV. Bats maintained at ambient temperatures were bled 60 and 180 days after the challenge. Hibernated bats were bled 45 and 180 days following the challenge.

### Tissues

Multiple tissues were collected upon necropsy. Tissues were processed as previously described [9]. Virus isolation was attempted on all brown and white adipose tissue samples that were positive by real-time RT-PCR. Briefly, in 96-well plates, neuroblastoma cells, (NA1300) were grown to 75% confluence in growth media (Eagle's minimum essential medium supplemented with 10% FBS, 100x MEM essential Vitamin mix, L-glutamine, tryptose phosphate broth, 50 μg streptomycin, and 100 IU penicillin G per ml). Media was aspirated and 200ul of the tissue homogenate was added to the cells. The plates were returned to a moist incubator (34°C, 5%CO_2_) and gently rocked every 15 minutes. After one hour, warm growth media was added to the wells and the plate was returned to the incubator. After 3 days, the cells were trypsinized and passaged into 2 new 96-well plates. No cells were discarded. After three days of growth, one plate was fixed as previously described and cell infection was assessed [9]. The cells in the second plate were passaged as described above. Trypsinized cells were divided between two new plates: one for subsequent passage and a second to evaluate cell infection via fixation and staining by the DFA [9]. If cell infection was not present after the fourth passage, the sample was determined to be negative and plates were discarded.

### Oral Swabs

Following inoculation with RABV, oral swabs were collected twice a week as described previously [9]. RNA extraction was performed on the QIAsymphony SP module (Qiagen, Germany) using the QIAsymphony DSP Virus/Pathogen Mini Kit and off-board lysis complex 200 ul protocol. Lysis was performed in the rabies laboratory per manufacture’s recommendations so the virus would be safely inactivated (68°C for 15 minutes) prior to extraction. Due to the fact that the amount of rabies virus RNA in orals swabs may be limited, real time RT-qPCR was performed immediately following extraction, thereby avoiding a freeze thaw cycle. qScript^tm^ One-Step Fast qRT-PCR Kit, Low ROX^tm^ (Quanta Biosciences, Gaithersburg, MD) was used for the generation of cDNA and PCR. The AS module of the QIAsymphony was employed to aliquot master mix and template. Following template addition, real-time RT-PCR was performed on the ABI 7500 fast using the following cycling conditions: one cycle at 50°C for 5 min and 95°C for 30 seconds followed by 45 cycles at 95°C for 15 seconds and 50°C for 60 seconds [10].

Both real-time RT-PCR and conventional RT-PCR were used, the former to assess viral load in tissue and saliva, the latter to determine the infecting variant by Sanger sequencing.

## Results

### Summary of experimental design

This study is comprised of two main groups; the first group, comprised of 6 subgroups, was inoculated with one of three bat RABV, either IM or SC and maintained at ambient temperature for the duration of the experiment. The study design for the second group was similar to the first group but all bats were inoculated IM and placed in hibernation for 6 weeks. Following the 6 week hibernation, bats were maintained at ambient temperature. Bats were bled at various time points to evaluate the presence of anti-rabies neutralizing antibodies. The amount of viral RNA in tissues and oral swabs of bats that developed rabies was determined using real time RT-PCR.

### Silver hair bats and hibernation

Bat 6, (EfV2), became active the second night in the hibernation chamber and was active every night thereafter. The bat was removed five days following introduction into the chamber and was warm and aggressive upon removal from the chamber. The bat had lost 1 g in the time since placement in the chamber 5 days previously and promptly began to eat worms when returned to its home cage. The bat remained aggressive for 3 days following removal from the hibernation chamber but continued to eat well, gain weight, and presented no additional signs of rabies virus infection. On the 4^th^ day, behavior improved and rabies was no longer considered a differential. This bat continued to gain weight and did not develop rabies during the experiment. Returning the bat to the hibernation chamber was not considered an option as we were not confident the bat would hibernate and the process of returning the bat would disturb the other bats in the hibernation chamber.

A second bat, bat 2 (LnV1) became active 14 days into hibernation. The bat continued to be active and was removed from the chamber 18 days into hibernation. She was warm when removed from the chamber and had lost 1g, but was not aggressive. The bat was returned to her home cage and ate a large number of mealworms. She continually gained weight until approximately 22 days (27dpi) after removal from the hibernation chamber. Twenty-two days after removal from the hibernation chamber, she began to lose weight despite appearing healthy, and eating and drinking normally.

Twenty-eight days after removal from the hibernation chamber, bat 2 became ataxic. An oral swab taken the previous day, 27 days after removal from the hibernation chamber was positive for rabies on real time RT-PCR. The results of the oral swabs from day 27 were received on day 28 and the bat was euthanized. The DFA test was also positive for rabies (Table 1).

Bat 13 (MlV1) became active 19 days after placement in the hibernation chamber. This bat was removed after 23 days in the hibernation chamber, was closely monitored, and remained healthy throughout the experiment.

The other 12 bats remained in hibernation for the full 6 week period. Bats aroused approximately every 10 days and were active for 60–90 min. Most bats aroused and returned to torpor within 10 min of each other.

### LnRV was the most virulent variant in its homologous species

Three of the five hibernated bats (60%) inoculated with LnV1 developed rabies. One bat inoculated with MlV1 and one bat inoculated with EfV2 developed rabies (Table 1).

In the ambient temperature study, five of the ten bats (50%) inoculated with LnV1 developed rabies (Table 2). Three of the five bats (60%) inoculated IM with LnV1 developed rabies whereas two of the five (40%) inoculated SC with LnV1 developed rabies. All bats inoculated IM with EfV2 survived. However, two of the five (40%) inoculated SC with EfV2 developed rabies. None of the bats inoculated with MlV1 via either the IM or SC route developed rabies. Although LnRV was the most virulent RABV in a homologous species, *L*. *noctivagans* was susceptible, albeit less so, to heterologous RABV.

### Hibernated and SC inoculated bats shed virus in their saliva and demonstrated increased incubation times

Regardless of the infecting variant, only bats that developed rabies following SC inoculation or were maintained in hibernation shed virus in their saliva (Tables 1 and 2). Viral RNA was not found in the saliva of bats that developed rabies following an IM inoculation and maintained at ambient temperatures.

Three of the five bats that developed rabies following hibernation shed viral RNA in their saliva. A positive oral swab was collected from bat 8 inoculated with EfV2 137 dpi, 90 days following removal from the hibernation chamber. Bat 4 (LnV1), began shedding virus 80 dpi, 33 days following removal from the hibernation chamber. A positive oral swab was collected from bat 2 (LnV1) 50 dpi, 27 days following removal from the hibernation chamber. The two bats that did not have viral RNA in their saliva, bats 3 and 11, had an overall longer incubation period from the time of inoculation 66 dpi and 70dpi, but a shorter time, 19d and 23d at ambient temperature following arousal, respectively (Tables 1 and 3).

**Table 3:** Dissemination of Rabies virus RNA evaluated by real time RT-PCR in hibernated bats. Data is provided as Ct values.

Bat ID	2	3	4	8	11	
Infecting variant/Route	LnV1/IM	LnV1/IM	LnV1/IM	EfV2/IM	MlV1/IM	
Days in hibernation	18	42	42	42	42	
Days at ambient temps following hibernation	27	19	33	90	23	No of positive specimens/total no tested (%)
Total incubation period dpi	50	66	80	137	70	
Tissue^a^						
SG	28.3	<LOD	28.5	21.8	<LOD	3/5 (60%)
Nose	31.7	35	33.4	29.0	35.5	5/5 (100%)
Tongue	29.3	<LOD	33.4	28.3	<LOD	3/5 (60%)
Diaphragm	34.3	<LOD	<LOD	27.3	<LOD	2/5 (40%)
Bladder	30.0	<LOD	<LOD	27.8	<LOD	2/5 (40%)
R. B plexus	28.3	27.8	26.4	18.3	27.4	5/5 (100%)
L.B plexus	26.4	<LOD	33.6	20.0	27.8	4/5 (80%)
R Sciatic N	30.3	33.6	31.1	28.5	32	5/5 (100%)
L. Sciatic N	31.0	<LOD	29.6	23.9	32	4/5 (80%)
GI	29.6	<LOD	28.9	23.7	<LOD	3/5 (60%)
Anterior SC	17.6	22.5	20.7	17.2	16.9	5/5 (100%)
Posterior SC	27.8	33.5	25.1	28.5	24.8	5/5 (100%)
Buccal tissue	<LOD^b^	<LOD	32.8	26.1	<LOD	3/5 (60%)
Inoculation site, muscle	31.7	30.7	23.9	30.7	<LOD	4/5 (80%)
Skin superficial to inoculation site	31.8	<LOD	<LOD	26.9	26.9	3/5 (60%)
Brown fat	17.6	36.6	30.0	17.2	30.0	5/5 (100%)
White back fat	29.6	34.5	39.6	28.5	<LOD	4/5 (80%)
Number of RV positive tissues / total tissues (%) +itive	16/17 (94%)	8/17 (47%)	14/17 (82%)	17/17 (100%)	9/17 (53%)	

^a^ SG, Salivary glands; B plexus, Brachial plexus; R, Right; L, left; N, Nerve GI, Gastrointestinal; SC, Spinal cord

^b^ LOD, results are below the limit of detection for our test

Of the bats maintained at ambient temperatures, viral RNA was detected in the oral swabs of two bats inoculated SC the same day clinical signs were first observed; bat 24 (SC, LnV1) and bat 35(SC, EfV2). Viral shedding was identified in bat 21 (SC, LnV1) and bat 32 (SC, EfV2) three days prior to the onset of clinical signs. There was no significant difference between the subcutaneously inoculated LnV1 and EfV2 groups. There was a significant difference in incubation times between bats that developed rabies following maintenance at ambient temperatures and hibernated bats. The extended incubation demonstrated in hibernated and SC inoculated bats increased the likelihood of viral shedding in the saliva and thus increased risk of transmission.

### Not all bats seroconverted following the first inoculation

Bats that were hibernated were bled twice-following arousal, once at 2 weeks and again at 4 weeks. Of the 15 inoculated bats, 6 (40%) developed VNA.

Bats maintained at ambient temperatures were bled 12 weeks following the first inoculation. Serum from eleven of the thirty (37%) inoculated bats was positive for VNA. The titers ranged from 0.25 IU to 5.0 IU with a median of 1.3 IU. As seen in Table 2, the group of bats inoculated SC with EfV2 included the greatest number of seropositive bats (n = 3) and the bat with the highest titer (5.0 IU). Lack of seroconversion may be a result of the neurotropic nature of RABV and lack of exposure to the immune system.

### Viral dissemination occurred in all bats, regardless of RABV variant or incubation time

Immediately following euthanasia, bats were necropsied and multiple tissues were collected. Viral RNA was present in most of the tissues assayed but the amount widely varied (Tables 3 and 4). As expected, the anterior spinal cord (ASC) contained the highest amount of viral RNA. It is noteworthy that viral RNA was not detectable in the salivary glands of the three LnV1 IM inoculated bats maintained at ambient temperatures throughout the experiment. Viral RNA was present in the salivary glands in all four bats inoculated SC with either LnV1 or EfV2, although with high Ct values. Viral RNA was present in the salivary glands of 3 of the 5 hibernated bats, two inoculated with LnV1 and one inoculated with EfV2.

**Table 4:** Dissemination of Rabies virus RNA in bats maintained at ambient temperature, as evaluated by real time-rtPCR. Data is provided as Ct values.

	Bat ID no. (infecting variant, route of inoculation, and days post inoculation)								
Bat ID	Ln37	Ln 17	Ln 19	Ln 20	Ln 22	Ln 24	Ln 32	Ln 35	
Infecting variant/route	LnV1^b^	Ln/IM	LnV1/IM	LnV1/IM	LnV1/SC	LnV1/SC	LnV1/SC	LnV1/SC	
Incubation: days post inoculation	>150	22	20	14	14	33	33	37	
Tissue^a^									No of positive specimens/total no tested (%)
SG	17.0	≤LOD	≤LOD	≤LOD^c^	27.1	32	28.0	27.4	5/8 (63%)
Nose	29.3	34	≤LOD	≤LOD	30.6	34	33.4	34.9	6/8 (75%)
Tongue	30.00	31	≤LOD	≤LOD	≤LOD	≤LOD	29.8	31.5	4/8 (50%)
Lung	26.8	30	≤LOD	≤LOD	26.9	37.0	30.1	29.0	6/8 (75%)
Diaphragm	26.4	31	≤LOD	30.9	≤LOD	32.4	28.4	30.3	6/8 (75%)
R. B plexus	22.4	25	27	24.5	25.7	24.6	28.4	25.7	8/8 (100%)
L. B plexus	28.3	26	29	31.0	26.8	26.9	24.0	25.9	8/8 (100%)
R. Sciatic N	24.3	30	37	31.6	26.6	29.7	28.8	28.2	8/8 (100%)
L. Sciatic N	≤LOD	28	≤LOD	≤LOD	30.1	27.6	29.0	32.0	5/8 (63%)
GI	19.3	28	≤LOD	≤LOD	26.9	ND^d^	33.5	39.3	5/8 (63%)
Anterior SC	17.9	21	21	19.3	19.3	19.1	20.6	18.3	8/8 (100%)
Posterior SC	21.0	25	≤LOD	30.3	21.7	34.0	30.4	24.4	7/8 (88%)
Buccal tissue	34.4	31	≤LOD	≤LOD	32.3	≤LOD	33.0	27.4	5/8 (63%)
Nuchal skin	26.8	27	28	32.4	34.7	24.8	25.7	24.5	8/8 (100%)
Inoculation site, muscle		28	30	≤LOD	≤LOD	28.8	26.7	30.3	5/7 (71%)
Inoculation site, skin		32	27	≤LOD	32.9	35.4	27.7	31.5	6/7 (86%)
Spleen	33.0	31	≤LOD	≤LOD	31.8	ND	≤LOD	30.4	4/7 (57%)
Brown fat	16.0	≤LOD	≤LOD	32.4	25.7	30.5	21.9	29.0	6/8 (75%)
White fat	25.8	31.3	≤LOD	≤LOD	≤LOD	≤LOD	30.6	26.5	4/8 (50%)
No. of positive tissues/total no. tested (%)	16/17 (94%)	17/19 (89%)	7/19 (37%)	8/19 (42%)	15/19 (79%)	14/17 (82%)	18/19 (95%)	19/19 (100%)	

^a^ SG, Salivary glands; B plexus, Brachial plexus; R, Right; L, Left. N, Nerve; GI, Gastrointestinal; SC, Spinal cord

^b^ This bat was caught150 days or more before clinical signs developed. Site of inoculation unknown

^c^ LOD, results are below the limit of detection for our test

^d^ Tissues not available for testing

Although viral RNA may appear to be associated with non-neuronal tissues, rabies antigen was limited to neuronal tissues (brain, spinal cord), brown adipose tissue, salivary tissue, and nerves innervating organs (data not shown). Given that the bats were inoculated in the right deltoid muscle or the subcutaneous tissue immediate superior to the right deltoid muscle, we evaluated the tissues collected from the right vs left side of the animal yet no significant difference was found. To investigate the difference in viral RNA loads between areas closer to the inoculation site versus more caudal locations, we compared the brachial plexuses and sciatic nerves; again, no significant difference was found. Considerable dissemination of viral RNA occurred in bats that developed rabies, regardless of RABV or inoculation route.

### Viral RNA was found in brown and white adipose tissue in experimentally infected bats

Samples of white and brown fat were collected from each bat during necropsy. In bats maintained at ambient temperatures, rabies RNA was present in a greater number of brown fat samples than white fat, 75% and 50% respectively. The Ct values of the brown fat samples ranged from 22 to >LOD. The Ct values in the white fat samples ranged from 25.8 to >LOD. In hibernated bats, 100% of brown fat and 80% of white fat tissue samples were positive, with Ct values ranging from 17.2 to 36.6 in brown fat and 28.5 to > LOD in white fat.

Virus isolation in cell culture was attempted for all positive brown and white fat samples, but was largely unsuccessful due to a high level of cell toxicity seen in cells inoculated with the adipose homogenates. To dilute the toxic effects, the homogenates were inoculated into larger volumes of media. This also resulted in a negative culture, possibly the effect of over dilution, lack of infectious virus, and/or inhibitors in the adipose tissue. However, based on positive Ct values, adipose tissue may provide a site for RABV maintenance.

### All bats survived the second inoculation

Nine months following the first inoculation, all surviving bats were challenged IM in the right deltoid with LnV1. Regardless of the route or RABV employed during the first inoculation, all bats survived. Despite two known exposures to rabies virus (1° and 2° inoculations), many bats did not seroconvert following the second inoculation (Tables 1 and 2). Five (50%) of the hibernated bats developed VNA. All five bats that developed VNA following the challenge had first been inoculated with a heterologous RABV. In the ambient temperature groups, fourteen of the twenty bats challenged (70%) did not develop detectable levels of VNA following the second inoculation. Similar to the hibernation study, all of the six bats that developed VNA had been exposed to a non-homologous RABV during the first inoculation. The development of VNA following inoculation with a heterologous RABV suggests heterologous RABV are more immunogenic than a homologous RABV.

### Naturally infected bat

The day following the first inoculation, 150 days following capture, one bat became severely ataxic, anorexic, obtunded, and exhibited unusual vocalizations. This bat was euthanized and was positive for rabies via the DFA. Conventional RT-PCR and Sanger sequencing results confirmed the infecting variant to be a *L*. *noctivagans* RABV. Pre-inoculation serum was negative for VNA but a sample obtained at the time of euthanasia demonstrated a neutralizing antibody response of 1.25 IU. Several tissues were collected at necropsy and viral RNA was found in most samples (Tables 2 and 4). Interestingly, the Ct values of most tissues were lower than samples collected from experimentally inoculated bats, suggesting greater amounts of virus were present in the tissues of the naturally infected bat as compared to the experimentally infected bats. Although previous attempts at isolating virus from the brown or white fat were unsuccessful, virus was isolated from the brown fat of the naturally infected bat after two passages in neuroblastoma cells. Virus could not be isolated from the white fat obtained from the naturally infected bat.

If this bat was exposed the day prior to capture, the minimum incubation time of the naturally infected bat would have been 5 months (151 days). If the bat aroused from torpor in early April and was caught 5 months later, the incubation time could be anywhere from 5–10 months. This incubation period is greater than any of the bats experimentally inoculated in our experiment. A longer incubation may be common in naturally infected bats due to the location of exposure, amount or titer of inoculum, components in the saliva that may affect transmission, and other factors unique to transmission in the wild.

### Hibernation extended the incubation period

None of the bats died or developed rabies during hibernation, yet the incubation period for all bats that developed rabies following hibernation was longer than bats maintained at ambient temperatures. For hibernated bats, the median incubation period from the day of inoculation (0 dpi) was 70 days with a range from 50–137 dpi. The median incubation period after removal from the hibernation chamber was 27 days with a range of 19–90 dpi (Table 1). With the exception of hibernated bat 8 (EfV2), the incubation times following removal from the hibernation chamber were similar to bats maintained at ambient temperature. The ambient temperature bats had a median incubation period of 22 days, with a range of 14–37 dpi (Table 2). There was a significant difference between the total incubation times of bats maintained at ambient temperature compared to hibernated bats (P < 0.05). The difference in incubation times is likely the result of decreased cellular metabolism and viral replication in the torpid bat.

### Bats regained weight soon after awakening

**Fig 1:**
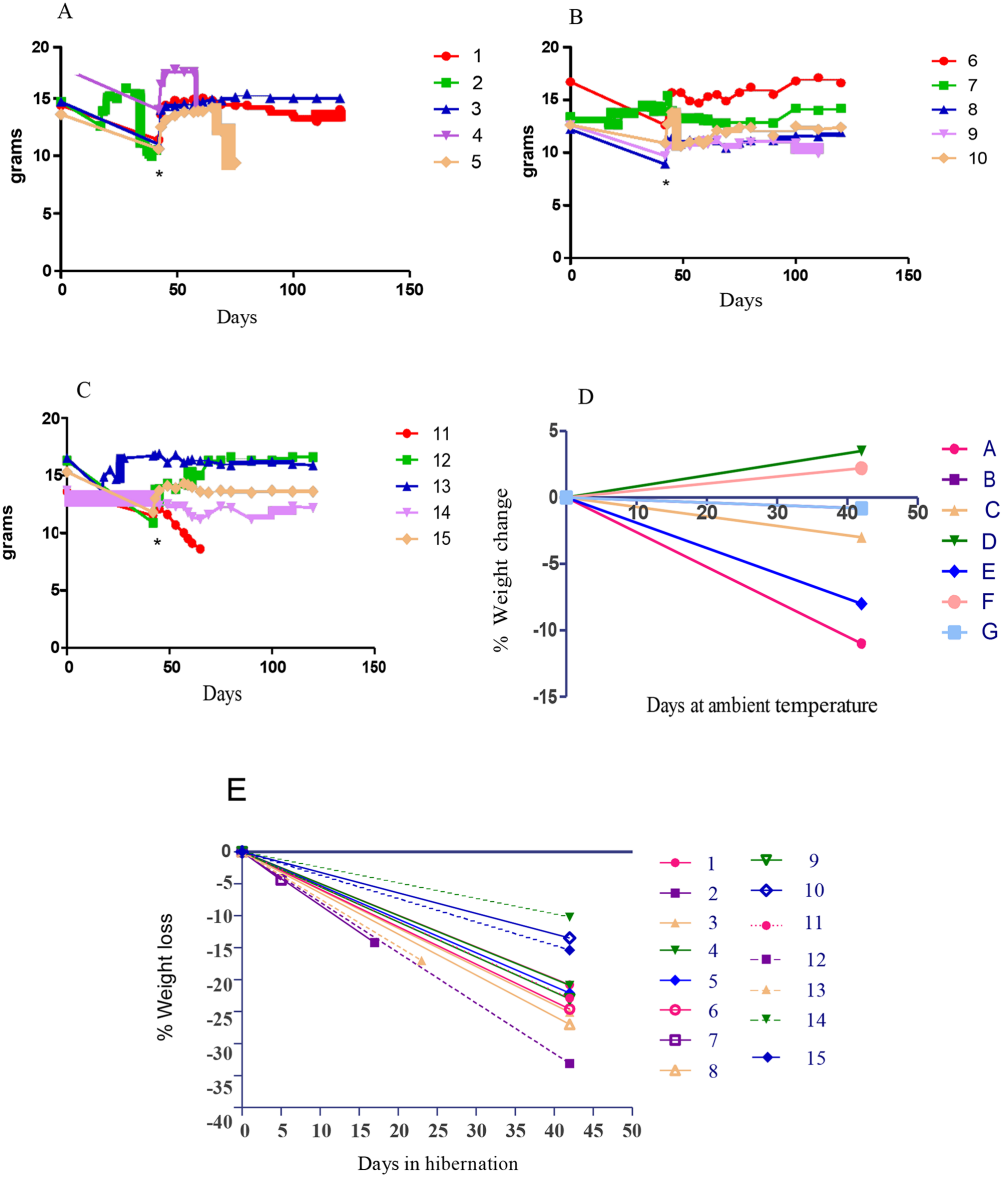
Weight changes in rabies inoculated bats following hibernation. Weight loss in hibernated bats inoculated with: A. LnV1. Bats 2, 3, and 4 developed rabies. B. EfV2. Bat 8 developed rabies. C. MlV1. Bat 11 developed rabies. * Denotes day 42. D. Percent weight change (grams) in non-inoculated control bats. E Percent weight change (grams) over the 6 week hibernation period.

Following arousal, bats were weighed then returned to their home cage and provided ad lib food and water. All bats that made it through the full 6 weeks appeared in good health upon removal from the hibernation chamber. Bats weights were monitored daily for the first 7 days to ensure health and weight gain. If bats continued to gain weight after the first 7 days, biweekly weighing was sufficient. Following hibernation, bats lost a median of 22% body weight, with an average of loss of 3.2g over 6 weeks. Post hibernation weight gain was rapid and most bats returned to pre hibernation weight within 1–2 weeks (Fig 1).

### Healthy bats are not determinately affected by rabid bats in neighboring cages

Rabid animals may exhibit unusual behavior such as movement disorders or vocalizations that may be perceived by healthy animals as a sign of illness [11–14]. If healthy bats are capable of sensing subclinical rabies infections in bats prior to overt clinical infection and eschew contact, this type of avoidance behavior may help explain the low percentage of rabid bats in a colony.

**Fig 2:**
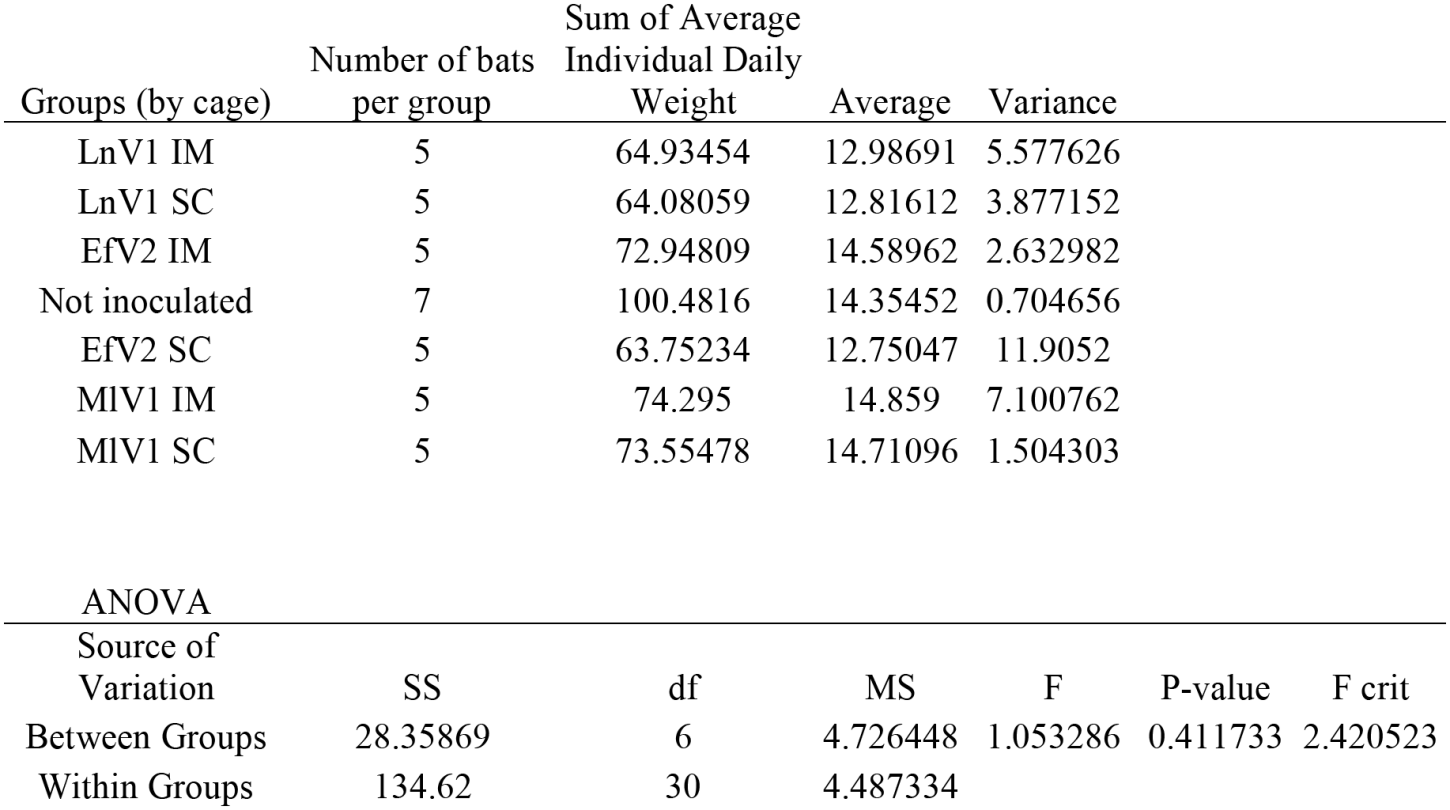
Single factor ANOVA of daily individual weight average grouped by cage reveals no variation in weight dependent on cage location.

Bats maintained at ambient temperatures were housed in groups of 5 based on the route and virus used in the inoculation. A group of 7 un-inoculated bats were included as controls. Although the groups were housed in separate cages, certain activities such as unusual vocalizations, fighting, and activity during daylight hours could be discerned among neighboring cages. To assess the impact of rabid bats on bats within the same cage and bats in neighboring cages, we examined the weights of bats before, during, and after a bat in the colony developed rabies. Bats exposed to stressors in their cages, such as unusual noises, cage mates that were active during the day, and aggression, were expected to demonstrate greater weight loss than bats in a well-controlled environment. However, no significant difference was found among groups (Fig 2).

## Discussion

Previous studies have suggested that the *L*. *noctivagans* RABV may be more virulent, yet it is unknown if *L*. *noctivagans* bats have an altered susceptibility to homologous RABV thereby increasing the number of rabid *L*. *noctivagans* in the wild populations as compared to *E*. *fuscus* and *M*. *lucifugus* [15–19]. If the answer was dependent on percentage of rabies positive *L*. *noctivagans* submitted to the NYSDOH rabies laboratory, the data would imply that tree bats, including *L*. *noctivagans*, had an increased susceptibility to rabies when compared to the commensal species *E*. *fuscus* and *M*. *lucifugus*. Over the past 25 years, an average of 17%, 4.0%, and 5.2% of the *Lasiurus cinereus* (Hoary bat), *L*. *noctivagans*, and *Lasiurus borealis* (Eastern red bat), respectively, submitted for the NYSDOH rabies laboratory for testing were positive for rabies virus. Conversely, the percentage of *E*. *fuscus* and *M*. *lucifugus*, 2.8% and 1.5% respectively, were positive for rabies [1]. The greater positivity reported in tree bats may be due to the small numbers submitted to the laboratory and their avoidance of roosting in structures that are shared with humans, such as houses and barns [20].

Based on data compiled from the NYSDOH rabies laboratory, the majority of rabid bats are submitted during late summer and early fall months [1]. The number of rabid bat submission sharply increases in May and June, one to two months following arousal from torpor and peaks in July, August, and the first week of September. This data suggests transmission occurs soon after arousal from hibernation continuing through the fall mating swarms, and possibly during hibernation based on incubation times seen in this study.

Bats maintained at ambient temperatures, regardless of inoculation route, developed rabies after significantly shorter incubations periods than bats that were hibernated. However, if one subtracts the time in hibernation from the overall incubation time, the difference is removed. Based on our results, we hypothesize that rabies virus remains latent during hibernation and is recrudescent when bat are moved to ambient temperatures. Similar to previous studies, a homologous RABV was most pathogenic, regardless if bats were maintained at ambient temperatures or torpor [8,9].

Sadler and Enright (1959) and Sulkin et al (1960) placed rabies inoculated bats in hibernation for varying lengths of time [21,22]. Bats were euthanized at certain time points, including during torpor. In the Sadler and Enright (1959) study, none of the bats euthanized during torpor were rabies positives [21]. However, Sulkin et al (1960) reported a small number of bats were rabies positive during hibernation [22]. Although both studies relied on the mouse inoculation test as opposed to the DFA to diagnose rabies, viral latency of rabies during torpor was clearly demonstrated. Sulkin et al (1960) reported several bats in which the salivary gland and brown fat were infected but the brain was not. These finding were not supported by our experiment. Based on the toxicity of brown fat in cell culture in our experiment, it is possible IC inoculation of mice with brown fat resulted in neurological manifestation that resembled rabies.

Although our study was similar in design and outcome to previous research, our study revealed several novel findings. We inoculated bats with three different bat variants and demonstrated no difference in incubation period between variants yet the homologous RABV was most virulent. Additionally, all bats in our study were hibernated at the appropriate time of years, as opposed to hibernating during April or May. Based on work by F. Griser, bats can only be truly hibernated in experimental settings at time when hibernation would occur in nature [23]. Forced hibernation can result in thermally stressing the animal, effecting experimental results. Moreover, the inclusion of advanced technology, including a well-controlled hibernation environment and real time RT-PCR advanced our understanding of hibernation and viral dissemination in insectivorous bats.

Earlier studies following the experimental inoculation of *E*. *fuscus* bats with St. Louis Encephalitis and Japanese Encephalitis viruses found that bats developed viremia within 10 days of removal from hibernation [24,25]. In the LaMotte study, bats were bled during several time points during hibernation and never developed viremia [24]. In animals maintained at ambient temperature viremia usually occurs from 2 to 5 days post inoculation [26]. Based on the data obtained from the current and previous studies, latency is a mechanism to ensure viral survival during times of year when the potential for transmission would be substantially decreased.

In both of our ambient temperature and hibernation experiments, less than half of the bats developed VNA following inoculation. Bats that were inoculated with a heterologous RABV were more likely to be seropositive and have higher titers. The differences may be explained by the increased immunogenicity of heterologous RABV.

Overall, viral dissemination was greater in bats with longer incubation times. In the hibernation experiment, the time spent at ambient temperatures following removal from the hibernation chamber was more important for viral dissemination that total incubation time. In bats inoculated SC, viral movement to the brain may be delayed as additional neural connections are required for CNS infection as compared with IM inoculation. Additionally, there may be selective pressure on the virus inoculated into the epithelial and connective SC tissues that is not found following IM inoculation. Although rabies must reach the brain prior to dissemination, less virus may reach the CNS following SC inoculation, allowing for viral dissemination prior to demonstrable clinical illness [27].

Viral dissemination among bats with longer incubation periods, such as the SC and hibernation groups, resulted in a greater likelihood of viral dissemination to salivary glands and thus increased potential for transmission. Based on our results, it is likely that rabies transmission among bats following hibernation may be due to IM or SC exposure from bites or grooming. However, in bats that do not undergo torpor, transmission is likely the result of a SC exposure occurring in mid-summer to late fall. Viral RNA was found in the brown fat of all hibernated and SC inoculated bats, and one of three (33%) bats inoculated IM. The presence of virus in the brown fat of bats may be a mechanism of viral maintenance.

Following removal from the hibernation chamber, bats quickly regained weight. Bats in hibernation become active when in distress, which could occur due to illness or starvation as seen in bats infected with White Nose Syndrome [7]. However, bats that roused prior to the end of the study began to eat immediately when returning to their home cage. *L*. *noctivagans* are migratory bats that hibernate, but unlike *E*. *fuscus* and *M*. *lucifugus*, they are not considered true hibernators. Bats that roused early from hibernation may have been younger bats or spent previous winters in areas in which long bouts of hibernation were unnecessary.

The reasons for the over- representation of *L*. *noctivagans* RABV in human infections are unclear [12,28]. *L*. *noctivagans* are less susceptible to a homologous RABV than *E*. *fuscus*, suggesting *L*. *noctivagans* are not contracting rabies at an increased rate over one of the most common commensal bats [8]. Viral dissemination and shedding are similar between *M*. *lucifugus*, *E*. *fuscus*, and *L*. *noctivagans*; therefore, *L*. *noctivagans* is not likely to transmit at a greater frequency than *M*. *lucifugus* or *E*. *fuscus*. The number of *L*. *noctivagans* that develop rabies following hibernation is similar to a previous hibernation study with *E*. *fuscus* in our laboratory (unpublished data). However, the lack of aggressive behavior demonstrated by clinically rabid *L*. *noctivagans* may increase the likelihood of a scratch or minor bite going unnoticed. In summary, the frequency of infection, shedding, and dissemination in *L*. *noctivagans*, as compared to *M*. *lucifugus* and *E*. *fuscus*, suggests the discrepancy of human rabies cases may be due to increased infectivity in heterospecific hosts, human susceptibility, and/ or behavioral factors.
